# Q&A: Extinctions and the impact of *Homo sapiens*

**DOI:** 10.1186/1741-7007-10-106

**Published:** 2012-12-20

**Authors:** Robert M May

**Affiliations:** 1 Department of Zoology, University of Oxford, The Tinbergen Building, South Parks Road, Oxford OX1 3PS, UK

## Extinctions have happened ever since life began - is there anything different about man-made extinctions?

Looked at in the large, the history of life on Earth is one of continuous change, driven by the interplay between evolutionary processes and the altered environments that can result. Some of these environmental events have had external causes (for example, the asteroidal impact that caused the most recent of the so-called Big Five mass extinctions, which eliminated the dinosaurs), while others have arisen from changing interactions among species (for example, the early appearance of oxygen in the atmosphere, resulting essentially from biogeochemical processes in primitive ecosystems). Are the recent past and impending future extinctions, unambiguously caused by humans, different? Yes and no. No, in the sense that the explosive growth of the animal species *Homo sapiens *can be seen as just another evolutionary process with increasingly serious ecological consequences for other species. Yes, in the sense that - unlike earlier extinctions - the causative agent (that's us) is aware of what is happening and could act to reverse current trends. Unfortunately, we show few signs of doing so.

## What are the major causes of extinctions (man-made or otherwise)?

The causes of recent, human-associated extinctions are usually listed under three headings: over-exploitation, habitat destruction, introduced aliens. But you could, with a bit of a stretch, brigade many past extinctions under one or more of these headings. The above-mentioned demise of the dinosaurs, or the massive wave of marine extinctions which mark the end of the Mesozoic, could be called 'habitat change'. The opening and closing of land bridges, as tectonic plates moved around over the past billion years and more, introduced 'invasive aliens', which restructured many ecosystems. More generally, over geological time-scales, natural evolutionary processes created changes within plant and animal populations, with new winners and new losers. In that sense, humans look like being the main agents of the Big Sixth wave of extinctions, on whose breaking tip we currently stand.

## How are the man-made versions distinct?

The very big difference between past extinctions and the current human-associated ones is we understand what is happening. And we can, in principle, choose to modify our behavior to preserve the awe-inspiring diversity of plant and animal life we have inherited. Even were we to do this - and we show few signs of it - there would still, over relatively long time-scales, be changes. They would, however, be more likely to be the pseudo-extinctions technically referred to as 'relay and replacement', as in the series of differently named species along the continuum as *Eohippus *evolved into today's horse.

## How much do we know about the rate of extinction before humans started interfering?

**Figure 1:**
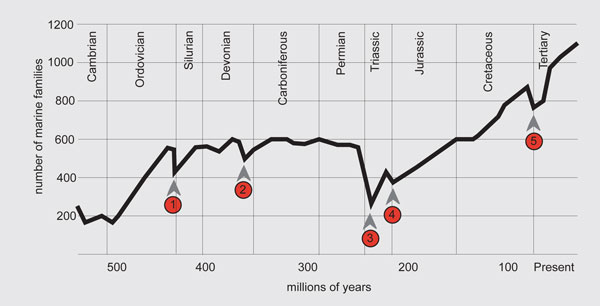
**The history of the diversity of the marine animal families over the past 600 million years**. The solid line connects 77 data points, each showing the total number of well-skeletonized families known from a particular geological epoch (each of whose duration is indicated by the width along the × -xis). The numbered arrows indicate the five recognized episodes of 'mass extinction'; the one labeled 3 is that which separates the Paleozoic from the Mesozoic, and the one labeled 5 is the one that ended the dinosaurs and ushered in our own era.

As in so many areas of science, we know quite a lot, and continue to learn more. One measure of our knowledge is indicated in Table 1, by Raup, which gives the estimated average lifetime, from origination to extinction, of a variety of animal groups. Figure 1 complements this by showing numbers of families (remember the taxonomic hierarchy: species, genus, family,...) of marine animals over the sweep of geological time. The figure testifies to increasing diversity and species richness, interrupted by episodes of mass extinction. Overall, these data suggest average life-spans of animal species in the fossil record to be around 1 to 10 million years, with significant variation within and among taxonomic groups, and with the higher end of the range being more common.

**Table 1:** Estimates of species' lifespan from origination to extinction

Taxon	Species' average lifespan (my)
All invertebrates	11

Marine invertebrates	5-10

Marine animals	4

Marine animals	5

All fossil groups	0.5-5

Mammals	1

Cenozoic mammals	1-2

Diatoms	8

Dinoflagellates	13

Planktonic foraminifera	7

Cenozoic bivalves	10

Echinoderms	6

Silurian graphtolites	2

## How much do we know about the rate of extinction after humans started interfering?

**Figure 2:**
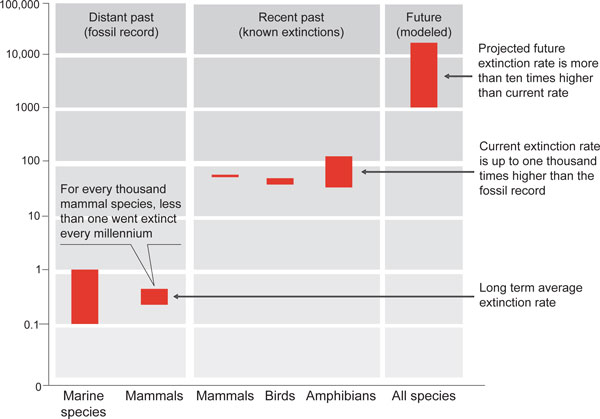
**Extinctions per thousand species per millennium**. This figure, taken from the Millennium Ecosystem Assessment, shows the estimated average lifetime of species in particular groups of animals, at different periods. 'Distant past' refers to average extinction rates as estimated from the fossil record. 'Recent past' is for extinction rates calculated from known extinctions of species (the lower estimate) or known extinctions plus 'possibly extinct' species (upper bound) over the past century or so. 'Future' extinctions are derived from a variety of different models, all based on current trends, but considerably uncertain (as indicated by the wider range).

Here our knowledge should be better. Part of the problem is what a management consultant might call the misallocation of resources. The workforce of systematists and taxonomists is estimated to be apportioned roughly equally among vertebrate animals, invertebrate animals and plants (with microorganisms an order of magnitude smaller). Yet the known number of vertebrate species is smaller than those of plant species and invertebrate species by one and two orders of magnitude, respectively. Things get worse as we move to research literature on conservation biology: a recent study of 2,700 papers published over 15 years in the two top conservation research journals shows 69% on vertebrates (four-fifths of the 69% on birds and mammals), 20% on plants, and 11% on invertebrates (one-third of the 11% on Lepidoptera). Nevertheless, if we assume that documented extinctions among birds and mammals occur at a rate typical of other groups, we can make an indirect assessment of the recent acceleration in extinction rates. The IUCN Red Data Books document the extinction of roughly one bird or mammal species each year over the past century. This is, in effect, a group of around 1,400 species each playing a game of Russian Roulette with one bullet in a revolver with 1,400 chambers. On this basis, each can expect to survive around 1,000 years. In relation to the 1 to 10 million year expectation noted above, this represents a speeding-up of extinction rates by a factor 1,000 to 10,000. Figure 2 shows this in more detail.

## Do we even know enough about how many species there are today?

If the Star Ship 'Enterprise' were to land on Earth, what would be the first question the crew asked of our planet? I think it would be, how many distinct species are there here? I think they would be shocked by our ignorance. We do have very good knowledge of how many bird species there are. The International Ornithological Congress says 10,448, although some would argue plus or minus 500. The mammalian total is smaller, 5,000 give or take 10%. Plant species add up to around 300,000. There are approximately 1 million known insects, but the true number could be several times this. Adding other smaller taxons gives a total species count in the neighborhood of maybe 1.7 million, although unresolved synonyms - same species identified and named separately in different museum collections - may inflate this. Estimates of the true total, in my opinion, are in the plausible range of 3 to 8 million distinct eukaryotic species. In other words, we have documented only one half, maybe only one-fifth, of our planet's biological diversity.

## Why should we be concerned about extinctions?

I would distinguish three kinds of concern.

The first might be called narrowly utilitarian: the plant and animal species that are being extinguished could represent important genetic resources for tomorrow's biotech revolution. We are burning the books before we have read them. I think this is a weak argument, because tomorrow's advances in understanding the molecular machinery of life will, I believe, see us (for example) designing new drugs from the molecules up.

The second might be called broadly utilitarian: although the services provided by ecosystems, which are many and varied, are not taken into account in conventional measures of gross domestic product (GDP) , they nevertheless are very important to us (and insofar as they can be given a value, it is estimated to be roughly of the magnitude of the more conventional global GDP). The Millennium Ecosystem Assessment classifies these services under 24 headings, and finds that 15 of these are being degraded, 4 are improving, and 5 are such that we know too little to assess. Deplorable though this is, I believe we may be smart enough to survive in a biologically impoverished world. It would, however, be an unattractive world resembling that of the cult movie *Blade Runner*.

Which brings me to the third argument, which is that we have an ethical responsibility not to deprive tomorrow's world of its heritage. Aldo Leopold expressed it well, mourning the death of Martha, the last passenger pigeon: 'We grieve because no living man will see again the onrushing phalanx of victorious birds sweeping a path for Spring across the March skies, chasing the defeated Winter from all the woods and prairies.... Our grandfathers, who saw the glory of the fluttering hosts, were less well-housed, well-fed, well-clothed than we are. The strivings by which they bettered our lot are also those which deprived us of pigeons. Perhaps we now grieve because we are not sure, in our hearts, that we have gained by the exchange'.

## Need it be an exchange?

That's the question.

## Where can I go for more information?

See references [1-5].

### Books

Lawton JH, May RM: *Extinction Rates*. Oxford University Press; 1995.

Raven PH: *Nature and Human Society: The Quest for a Sustainable World*. Washington DC: National Academy Press; 1977.

Millenium Ecosystem Assessment: *Ecosystems and Human Well-being: Synthes*is. Washington, DC: Island Press; 2005.
